# Characteristic and expression of *Hsp*70 and *Hsp*90 genes from *Tyrophagus putrescentiae* and their response to thermal stress

**DOI:** 10.1038/s41598-021-91206-2

**Published:** 2021-06-03

**Authors:** Jing Wang, Sheng-quan Que, Xinyu Liu, Mengru Jin, Tian-Rong Xin, Zhi-wen Zou, Bin Xia

**Affiliations:** 1 School of Life Sciences, Nanchang University, Nanchang, 330031 China; 2 Institute of Forest Pest, Jiangxi Academy of Forestry, Nanchang, 330013 China

**Keywords:** Biological techniques, Expression systems, Functional genomics

## Abstract

The development of insects is critically affected by temperature, which therefore plays an important role in the control of stored grain pests. Extreme temperature stress conditions lead to biological responses in mites, such as the synthesis of heat shock proteins. *Tyrophagus putrescentiae* (*Tp*) is a pest mite in stored grain that has negative effects on both economy and health. Since *T. putrescentiae* population dynamics are strongly influenced by temperature, in the present study we have cloned the cDNA of *HSP70* and *HSP90* (referred to as *TpHSP70-1*, *TpHSP70-2* and *TpHSP90*) and determined their expression by fluorescence real time quantitative PCR. *TpHSP70* and *TpHSP90* showed high homology with similar genes in other species and the open reading frames of *TpHSP70-1, TpHSP70-2* and *TpHSP90* encoded proteins of 665, 661 and 718 amino acid residues, respectively. Under thermal stress, expression of *TpHsp70-1* and *TpHsp90* was up-regulated at higher temperatures, suggesting their role in the defense against thermal stress.

## Introduction

Storage mites are aeroallergens that can cause asthma and rhinitis in sensitised individuals^1,2^. In some cases, they may lead to anaphylaxis caused by the ingestion of contaminated food^3,4^. *Tyrophagus putrescentiae* is a mite that can be found worldwide in farms, laboratories, urban environments and food industries^5,6^ from where they have been successfully isolated^7,8^. *T. putrescentiae* is also associated to host bacterial communities or symbionts in the gut, fat body or other tissues^9–11^.

Normal growth and development in insects requires appropriate temperature, otherwise they stagnate and even die^12^. Their body temperature is close to that of their habitat, making them vulnerable to extreme temperatures^13^. High temperatures can cause developmental abnormalities or defects^12,14^. Since immature mites and other poikilotherms cannot efficiently regulate body temperature, the latter is usually the most important environmental factor that impacts in their development rate^12,15^.

In *T. putrescentiae,* the full normal pattern of development of early immature stages, egg, larva and protonymph, has been suggested to be seriously impaired under temperature stress and reproductive parameters and temperature are closely related^16,17^.

Heat shock proteins (Hsps) are molecular chaperones with crucial roles in protein folding and unfolding, aggregation, degradation and transport^18^. They are also important for the insect survival under thermal stress^17,19^. Indeed, when Drosophila was treated at 30–39 ℃, heat resistance correlated with *Hsp*70 gene expression at different stages^20^. When *Hsp70* gene expression was inhibited by RNAi in *Bemisia tabaci* female, survival was lower than in the control group^21^. The literature reports the study of *Hsp90* and *Hsp70* genes of a few mites in the Tetranychidae. In *Panonychus citri,* although the three Hsp70 proteins were expressed under cold shock, only *Hsp70-2* was up-regulated under heat shock, whereas *Hsp90* gene was expressed under high temperature stress, suggesting that both *Hsp90* and *Hsp70-2* proteins play an important role in the adaptation to high temperatures^22,23^. Also, in *Tetranychus cinnabarinus, Hsp70-1* and *Hsp70-3* play a vital role in cold and heat stress^24^. Overall, these results suggest that *Hsp*s play an important role in improving heat resistance of mites.

Herein, to understand the adaptation of *T. putrescentiae* to temperature stress we have cloned its full-length *Hsp*70 and *Hsp*90 cDNAs and measured their mRNA expression at different temperatures using real-time quantitative polymerase chain reaction (RT-qPCR).

## Materials and methods

### Mites

*T. putrescentiae* adults were collected from the storages at Nanchang suburb, Jiangxi Province, China. The mites were fed with wheat bran (Shangdong, China) in specially made plastic containers (18 × 11 × 8 cm) covered with a lid to prevent escape. A 3 cm diameter hole was shorn on the lid for ventilation^25^ *T. putrescentiae* of different ages were identified by our laboratory teachers using the Manual of Acarology 3rd Edition as a reference^25^. Climate-controlled incubators (RXZ-260B) were used to keep the rearing units for several generations at 25 ± 0.5 ℃ and 75 ± 5% relative humidity (controlled by a YADU ultrasonic humidifier) in the dark.

### Total RNA extraction and cDNA synthesis

300 female adults of *T. putrescentiae* were taken from the experimental population. Total RNA was extracted using the TRIzol method (Invitrogen, San Diego, CA, USA) and then treated with DNase I (Tiangen, Beijing, China)^23,26^. Concentration and purity of RNA was assessed with a NanoDrop2000 spectrometer (Thermo, USA) at 260 nm and 280 nm. Finally, the integrity of total RNA was tested using 1% agarose gel electrophoresis. The first strand cDNAs were obtained using the Reverse Transcription M-MLV Kit (TaKaRa, Tokyo, Japan): 1 µL of random 6-mers, 1 µL of dNTP mixture and 8 µL of total RNA were mixed and incubated at 65 ℃ for 5 min to improve reverse transcription efficiency. Then, 4 µL of 5 × PrimeScript II Buffer, 0.5 µL of RNase Inhibitor and 1 µL Primer Script II RTase were mixed with RNase-free water up to a final volume of 20 µL. Finally, the mixture was incubated at 45 ℃ for 50 min and at 70 ℃ for 15 min. The cDNA was stored at − 20 ℃ for subsequent experiments. Each sample was processed with three biological replicates.

### Degenerate primers and amplification of cDNA

To amplify partial cDNA fragments of *Hsp*70 and *Hsp*90, degenerate primers were designed (Table 1) and used in PCR as described previously^23,27^. PCR reactions used 0.1 μg cDNA as template, 0.3 μM of each primer, 12.5 μL 2 × Taq polymerase Mix (Tiangen, Beijing, China) and ddH_2_O was added to a total volume of 25 μL.

**Table 1:** Primers used in this study.

Experiments	Primer name	Primer sequences(5′–3′)	Primer size (bp)	Tm (℃)	GC%	Amplicon size (bp)
Full length PCR amplification	*TpHSP70-1*F	CAAGCTACCAAACGCGACTAACC	23	55	52	766
	*TpHSP70-1*R	AAAGGAGCCGCAATGTTGAATG	22	57	45.5	
	*TpHSP70-2*F	AAAGGAAGAGGTTGGCAAGGAA	22	56	45.5	768
	*TpHSP70-2*R	CAGTTGAAGGAAGGGTAGAGTGGA	24	55	50	
	*TpHSP90*F	TACTCTACGCTTTACATCCGAACAC	25	49	44	750
	*TpHSP90*R	GAACAACTTAATCAACCTCCTCCAT	25	49.5	40	
Hsp70-3'-RACE	*3TpHSP70-1*F1	CACCAAGATGAAGGAGACCGCCGAG	25	60	60	1741
	*3TpHSP70-1*F2	CAAGAAGACCCAGGGCGAGAAGAAC	25	59	56	
	*3TpHSP70-2*F1	ACAAGAAGGAGGGCGAGAAGAAC	23	57	52.2	1140
	*3TpHSP70-2*F2	GATCGACAATGAAGCCCGTGCAG	23	59	56.5	
Hsp70-5'-RACE	*5TpHSP70-1*R1	GATCGACTTGTTGAGCTCCTTGCC	24	58	54.2	1095
	*5TpHSP70-1*R2	GGCACGGGTGATCGAGGAGTAGAA	24	60	58.3	
	*5TpHSP70-2*R1	GCTGCGAATGTCCTTGCCGGTCT	23	61	60.9	890
	*5TpHSP70-2*R2	CCACCGCCCAGATCGAACACCAG	23	63	65.2	
Hsp90-3'-RACE	*3TpHSP90*F1	CGTCAAGAAGTGCCTGGAGCTGT	23	58.5	56.5	747
	*3TpHSP90*F2	TGAGGAGAAGAAGAAGCGCGAGG	23	58.5	56.5	
Hsp90-5'-RACE	*5TpHSP90*R1	CATGTAGACGACCTCGAAGCCAC	23	58.5	56.5	1402
	*5TpHSP90*R2	CGTCAAACAGCTCCAGGCACTTC	23	58.5	56.5	
Real-time PCR	*qTpHSP90*F	TTCCAGTGGCGACGAGATGT	20	60.8	55	180
	*qTpHSP90*R	TCCTCAGGCAGCTCCAGACC	20	62	65	
	*qTpHSP70-1*F	CACTAACGACAGGCGATTCTACG	23	59.4	52.2	150
	*qTpHSP70-1*R	TCAACCGAACAACCACTCAAGC	22	58.5	50	
	*qTpHSP70-2*F	GAACTGGTCCTGCTCGATGTGAAC	24	60.5	54.2	120
	*qTpHSP70-2*R	ACCCTCGAAGACTTGAATTGTGACG	25	57	48	
	*qTpTubulin*F	TATCCTCGCATTCACTTTCCTCTGG	25	57	48	100
	*qTpTubulin*R	CGAAGCAGGTGTTGGTAATCTCAGC	25	59	52	

The PCR programs were operated with the following cycling conditions: initial denaturation step of 3 min at 94 ℃ followed by 35 cycles of 94 ℃ (30 s), 49 ℃ (30 s), 72 ℃ (60 s) and 72 ℃ (10 min). PCR products were detected with 1% agarose gel. Bands with expected size were purified with a universal DNA purification kit. Purified DNA fragments were cloned into a pGEM-T Easy vector and transfected into *Escherichia coli* DH5α (Promega, Madison, WI, USA). DNA inserts of the recombinant clones were confirmed by PCR with the same degenerate primers used previously and by sequencing in both directions.

### Rapid amplification of cDNA ends

The rapid amplification of cDNA ends (RACE) method was applied to obtain full-length cDNAs. Gene specific primers were designed (Table 1) using the identified *Hsp*70 and *Hsp*90 cDNA fragments, and 5′ and 3′-Full RACE Kits (TaKaRa, Tokyo, Japan) were used to amplify the 5′ and 3′-ends of the two genes. The first-round PCR program was pre-denaturation at 94 ℃(180 s) followed by 35 cycles of 94 ℃ (30 s), 60 ℃(30 s) and 72 ℃ (90 s) with a final extension at 72 ℃ (6 min). The second-round PCR program was the same as the first-round.

The PCR products from the 5′- and 3′-RACE reactions were cloned into the pGEM-T Easy vector and transfected into *Escherichia coli* DH5α cells (Promega, Madison, WI, USA). Six recombinant clones were identified by PCR amplification and sequenced (Sangon, Shanghai, China).

### Confirmation of full-length cDNA sequences

After the 5′- and 3′-ends sequences were obtained, contigs were assembled with the Seqman software^28^ to produce the putative full length sequences of *HSP70* and *HSP90.* Full length cDNAs were verified by amplification of the ORFs using the primers listed in Table 1. PCR products were cloned into a pGEM-T Easy vector and sequenced. PCR conditions and cloning methods were as described above.

### Bioinformatics analysis

The sequences of *TpHSP70 and TpHSP90* were blasted at the National Center for Biotechnology Information website (http://www.ncbi.nlm.nih.gov/BLAST/) at both nucleotide and amino acid levels. Amino acid sequences were analyzed with the Expert Protein Analysis System (http://www.expasy.org/). Multiple alignments of *TpHSP70 and TpHSP90* were analyzed in the DNAstar software (7.1 version). Neighbor-joining phylogenic trees were constructed with ClustalX 2.0 and MEGA 5.0 using the gene sequences of HSP70 and HSP90 (sequences shown in Table 2). The confidence of the branches was obtained using 1000 replicates’ Bootstrap analysis.

**Table 2:** *HSP70* and *HSP90* sequences used in phylogenetic analysis.

Accession Number	Organism	Hsp70 or Hsp90
NP_001036837	*Bombyx mori*	*BmHSP70-1*
NP_727563	*Drosophila melanogaster*	*DmHSP70-3*
AIS39468	*Haemaphysalis lava*	*HlHsp70*
NP_005338	*Homo sapiens*	*HsHSP70*
XP_002407132	*Ixodes scapularis*	*IsHsp70-2*
XP_002433656	*Ixodes scapularis*	*IsHsp70-1*
ABK76338	*Marsupenaeus japonicus*	*MjHsp70*
BAE39187	*Mus musculus*	*MmHsp70-1*
BAE41082	*Mus musculus*	*MmHsp70-2*
AGQ50609	*Neoseiulus cucumeri*	*NcHsp70*
ADE34170	*Nilaparvata lugens*	*NlHsp70*
XP_001510947	*Ornithorhynchus anatinus*	*OaHsp70*
ADM83423.1	*Panonychus citri*	*PcHsp70-1*
ADM83424.1	*Panonychus citri*	*PcHsp70-2*
ADM83425.1	*Panonychus citri*	*PcHsp70-3*
NP_077327	*Rattus norvegicus*	*RnHsp70*
ACG60423	*Tetranychus cinnabarinus*	*TcHsp70-2*
ACG60424	*Tetranychus cinnabarinus*	*TcHsp70-3*
ABC33921	*Tetranychus urticae*	*TuHSP70*
AAB97092	*Xenopus laevis*	*XlHsp70*
KR479867	*Tyrophagus putrescentiae*	*TpHSP70-1*
KR479868	*Tyrophagus putrescentiae*	*TpHSP70-2*
AJQ31840.1	*Tyrophagus putrescentiae*	*TpHSP90*
NP_001036876.1	*Bombyx mori*	*BmHSP90*
ADP37710.1	*Helicoverpa armigera*	*HaHSP90*
BAE44307.1	*Chilo suppressalis*	*CsHSP90*
AAA96259.1	*Xenopus laeviens*	*XlHSP90*
CAA30276.1	*Drosophila melanogaster*	*DmHSP90*
ACO83357.1	*Penaeus monodon*	*PmHSP90*
EEC05121.1	*Ixodes scapularis*	*IsHSP90*
XP_003745497.1	*Metaseiulus occidentalis*	*MoHSP90*
AAB23369.1	*Rattus* sp*.*	*RaHSP90*
AAI21063.1	*Homo sapiens*	*HsHSP90*
BAK08840.1	*Chara braunii*	*CbHSP90*
AGQ50610.1	*Neoseiulus cucumeris*	*NcHSP90*
ACF75907.1	*Tetranychus cinnabarinus*	*TcHSP90*
ADM83426.1	*Panonychus citri*	*PcHSP90*

### mRNA expression of *TpHSP70 and TpHSP90* under thermal stress

#### Sample collection at different stages

The eggs of *T. putrescentiae* were separated with a 140 mesh *sieve*. These eggs continued to grow and develop and were observed every 12 h. After many generations, about 400 eggs, 400 larvae, 200 nymphs and 200 female adults were taken for total RNA extraction. Each experiment was repeated three times.

#### Thermal stress treatment

Two hundred female adult mites were transferred to 1.5 mL centrifuge tubes sealed with a 0.45 µm filter membrane (BBI, China) for ventilation. In total, ten tubes with 200 mites each were exposed to 0, 5, 10, 15, 20, 30, 33, 36, 39, and 42 ℃, respectively for 1 h, where mites kept at 25 ℃ were used as control group. Three replicates were used for each group.

### qPCR of *TpHSPs*

qPCR assays were performed on Fx960 Real-time Quantitative PCR (BIO-RAD, USA) with alpha-tubulin gene from *T. putrescentiae* (GenBank accession number: AY986760) as endogenous reference. Each PCR reaction was mixed with 10 µL TB green, 7.8 µL ddH_2_O, 1.0 µL cDNA, 0.4 µL Rox dye and 0.4 µL of each primer. The thermal cycling profile consisted of an initial denaturation at 95 ℃ (5 min) and 40 cycles at 95 ℃ (10 s) and 60 ℃ (20 s). For each gene specific primer, three independent replicates were performed, each repeated three times. The expression levels of *TpHSP70 and TpHSP90* were calculated with the 2^−ΔΔCt^ method^29^.

### Data analysis

Data was represented as the mean ± SE (standard error) for all data sets. The data were then subjected to a one-way analysis of variance (ANOVA) using SPSS 26.0 (Chicago, IL, USA). Differences between means were tested using the Duncan’s test for multiple comparisons. Differences were considered statistically significant at the 5% level (*p* < 0.05).

## Results

### Sequence analysis of *TpHSP70* and *TpHSP90* genes

**Figure 1:**
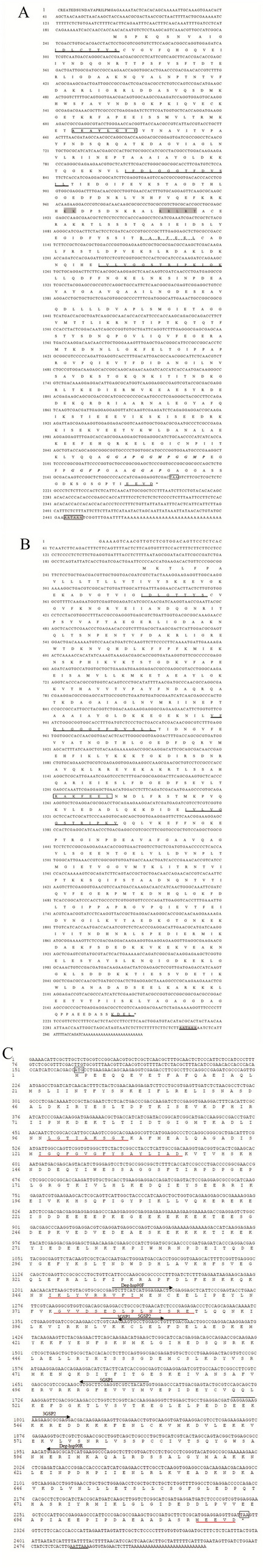
Nucleotide and deduced amino acid encoding region of *TpHSP70-1 *(**A**), *TpHSP70-2* (**B**) and *TpHSP90* (**C**). Signature sequences of *TpHSP* genes are marked with underline.

The complete cDNA sequences of *TpHSP*70-1 (GenBank accession number: KR479867) and *TpHSP*70-2 (GenBank accession number: KR479868) were 2494 (ORF of 1,998) and 2354 bp (ORF 1984 bp) long, respectively (Fig. 1A, B). The *TpHSP*70-1 cDNA included a 186 bp 5′ untranslated region (UTR) and a 310 bp 3′ UTR. *TpHSP*70-1 encodes a 665 amino acid protein with a calculated molecular weight of 72.72 kDa and an isoelectric point of 5.21. The *TpHSP*70-2 cDNA included a 168 bp 5′ untranslated region and a 173 bp 3′ UTR. *TpHSP*70-2 encodes a 661 amino acid protein with a calculated molecular weight of 72.75 kDa and an isoelectric point of 5.29. For both proteins, a possible consensus signal sequence for polyadenylation (AATAAA) was located 45 bp upstream of the poly (A) tail and both have three motifs typical of the Hsp70 proteins family.

The complete cDNA of the *TpHSP*90 gene was deposited in GenBank with accession number KJ820823 and consisted of 2538 bp with an ORF of 2157 bp, which encoded a 718 amino acid protein (Fig. 1C). The *TpHSP90* cDNA included a 165 bp 5′ untranslated region located upstream of the putative start codon (ATG) and a 216 bp 3′ UTR located downstream of the stop codon. The mature protein had a calculated molecular weight of 82.79 kDa with an isoelectric point of 4.92. A possible consensus signal sequence for polyadenylation (AATTAAA) was located 15 bp upstream of the poly (A) tail. The typical histidine kinase-like ATPase domain, ubiquitous in all *Hsp*90 family members, was located at the position of 37–181. *TpHSP*90 contained the five typical motifs observed in *Hsp*90 proteins: NKEIFLRELISNASDALDKIR, LGTIAKSGT, IGVFGVGFYSAYLIAD, IKLYVRRVFI and GVVDSEDLPLNISRE. The C-terminal "MEEVD" motif, which is specific to the of Hsp90 family (cytoplasmic type). Comparative analysis showed that the amino acids of HSP90 in *T. putrescentiae* presented high similarity of 79–81% with Hsp90 in other species.

### Homology analysis of *TpHSP*70 and *TpHSP*90

**Figure 2:**
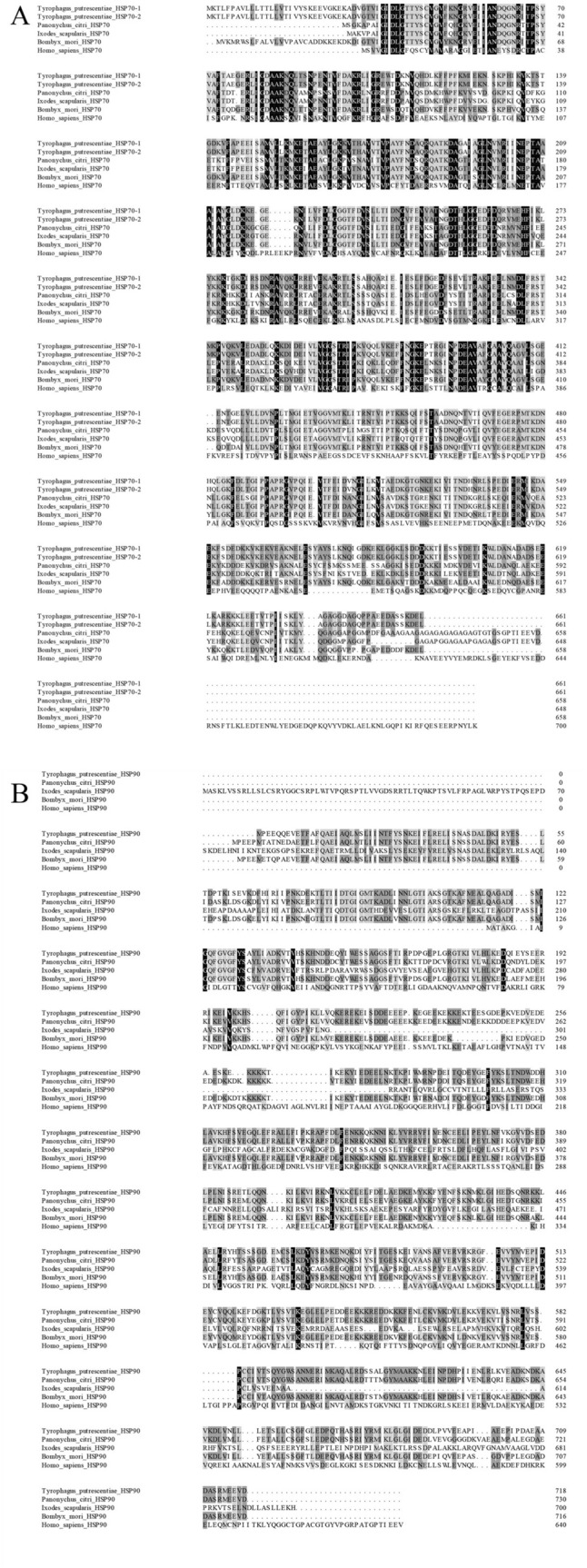
Comparison of the amino acid sequences of *HSP70* protein family (**A**) and *HSP90* protein family (**B**).

**Figure 3:**
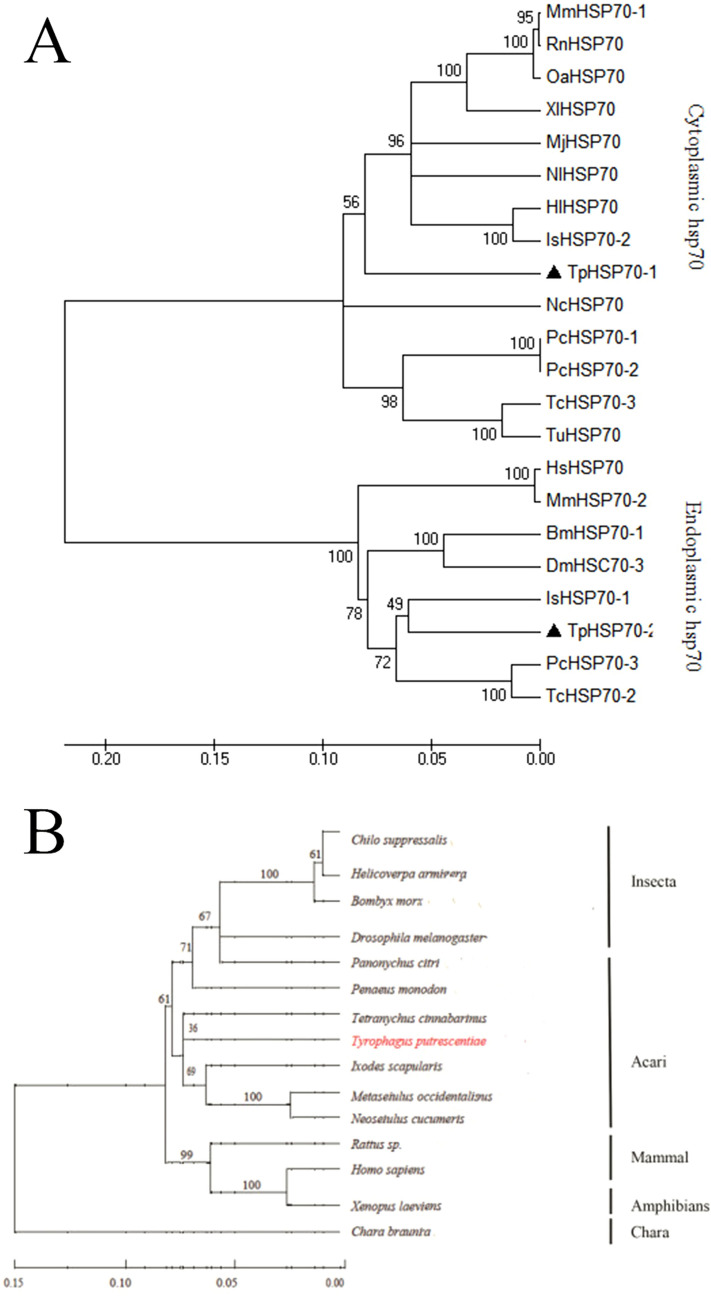
Phylogenetic tree of *HSP70* (**A**) and *HSP90* (**B**) from *T. putrescentiae* and other species. Constructed by the neighbor joining method based on amino acid sequences. Numbers at each branch indicate the percentage of times, and a node is supported in 1,000 bootstraps pseudo-replication by neighbor joining.

A BLASTP (http://blast.ncbi.nlm.nih.gov/Blast.cgi) search of GenBank revealed that *TpHSP70-1 and TpHSP70-2* belong to the *HSP70* family and that *TpHSP90* belongs to the *Hsp90* family. Multiple sequence alignments showed that the deduced amino acid sequences of *TpHSP70-1 and TpHSP70-2* share high similarity with three HSP70s from *Tetranychus cinnabarinus*, *Ixodes scapularis*, *Bombyx mori*, *Drosophila melanogaster* and *Homo sapiens* (Fig. 2A). Phylogenetic analyses showed that *TpHSP70-1* and *TpHSP70-2* belong to the cytoplasmic and the endoplasmic reticulum types of the phylogenetic tree, respectively (Fig. 3A).

The deduced amino acid sequence of *TpHSP*90 shared high similarity with five HSP90s from *T. cinnabarinus*, *I. scapularis*, *B. mori*, *D. melanogaster* and *H. sapiens* (Fig. 2B). Among these, *TpHSP*90 showed the highest similarity to *Hsp*90 from *I. scapularis* (81% identity), and lowest similarity with *HSP*90 from *D.melanogaster* (77% identity). *HSP*90 homology was high within the arthropods, especially in the signature regions of the *Hsp90* family. The relationships between *HSP90s* displayed in the phylogenic tree are consistent with the traditional taxonomy of these species (Fig. 3B). *HSP90* from *T. putrescentiae* and *T. cinnabarinus* cluster together earlier than *I. scapularis*, *M. occidentalis* and *N. cucumeris* (Fig. 3B).

### Expression of *TpHSP70* and *TpHSP90* in different stages of development

**Figure 4:**
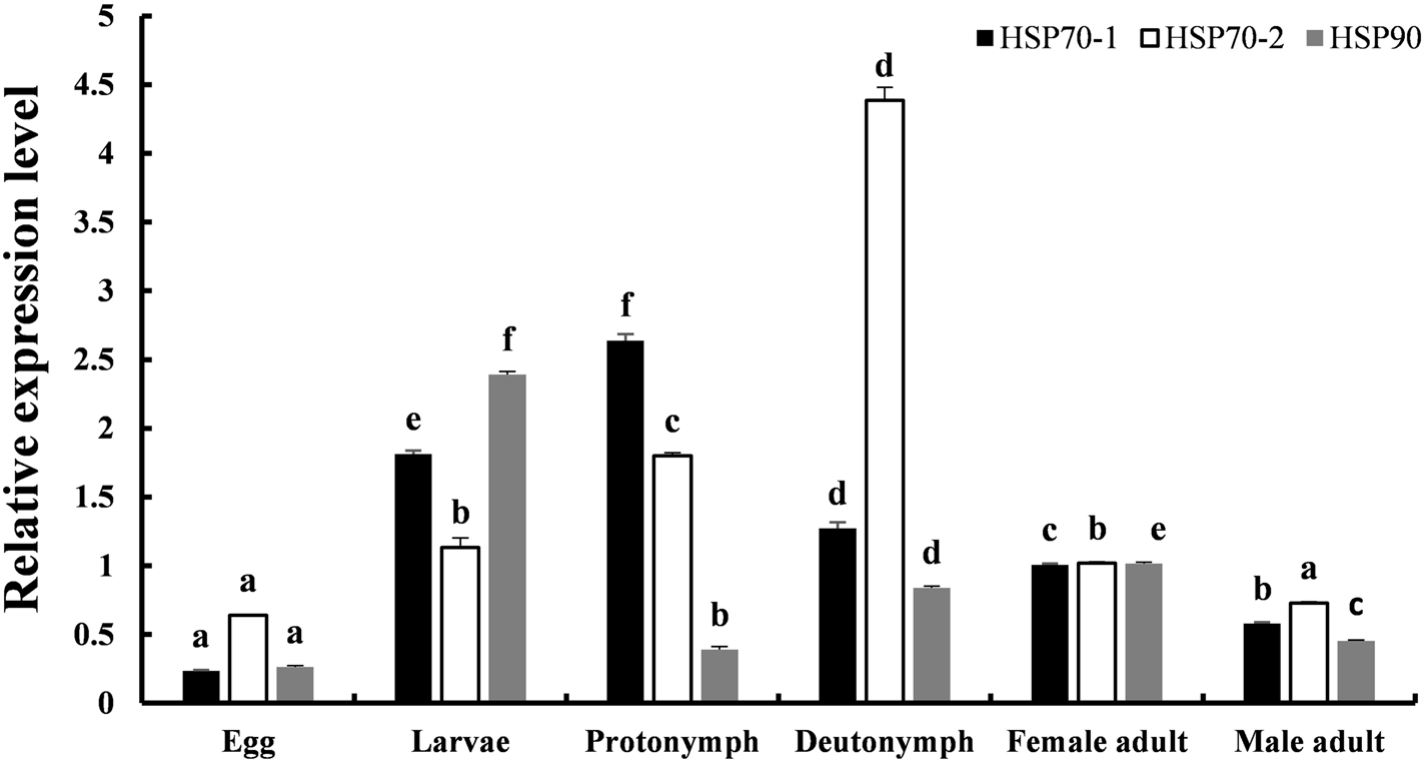
Stage-specific *TpHSP70-1*, *TpHSP70-2* and *TpHSP90* expression in *T.putrescentiae*. The mRNA expression level of *TpHSP70-1*, *TpHSP70-2* and *TpHSP90* genes in different developmental stages including egg, protonymph, deutonymph, tritonymph and adult stages of *T.putrescentiae* was measured by fluorescent real-time quantitative PCR. Values are the mean ± SD (n = 200). The different letters above the bars (**a**–**f**) indicate a significant difference in the means as assessed using Duncan’s multiple comparison tests (*P* < 0.05).

Tubulin was used as a reference gene to measure the mRNA expression level of *TpHSP90, TpHSP70-1* and *TpHSP70-2* in different developmental stages of *T. putrescentiae:* egg, larva, protonymph, tritonymph and adult. The mRNA expression levels of all three genes increased with the development of the mites, but their genes’ expression levels varied greatly (Fig. 4). Although the expression levels of the *TpHSP70-2* gene changed with developmental stage, no significant differences were observed in the expression levels of *TpHSP70-1* gene and *TpHSP90* genes across developmental stages. The expression level of *TpHSP70-1*, *TpHSP70-2* and *TpHSP90* genes was highest in the protonymph, tritonymph and in the larva, respectively. Thus overall expression of three *TpHSPs* was highest in the immature stages, and lower in the egg and adult stage. Interestingly, expression of heat shock protein genes was higher in female mites than in male mites.

### Dependency of *TpHSP*90 and *TpHSP*70 expression on temperature

**Figure 5:**
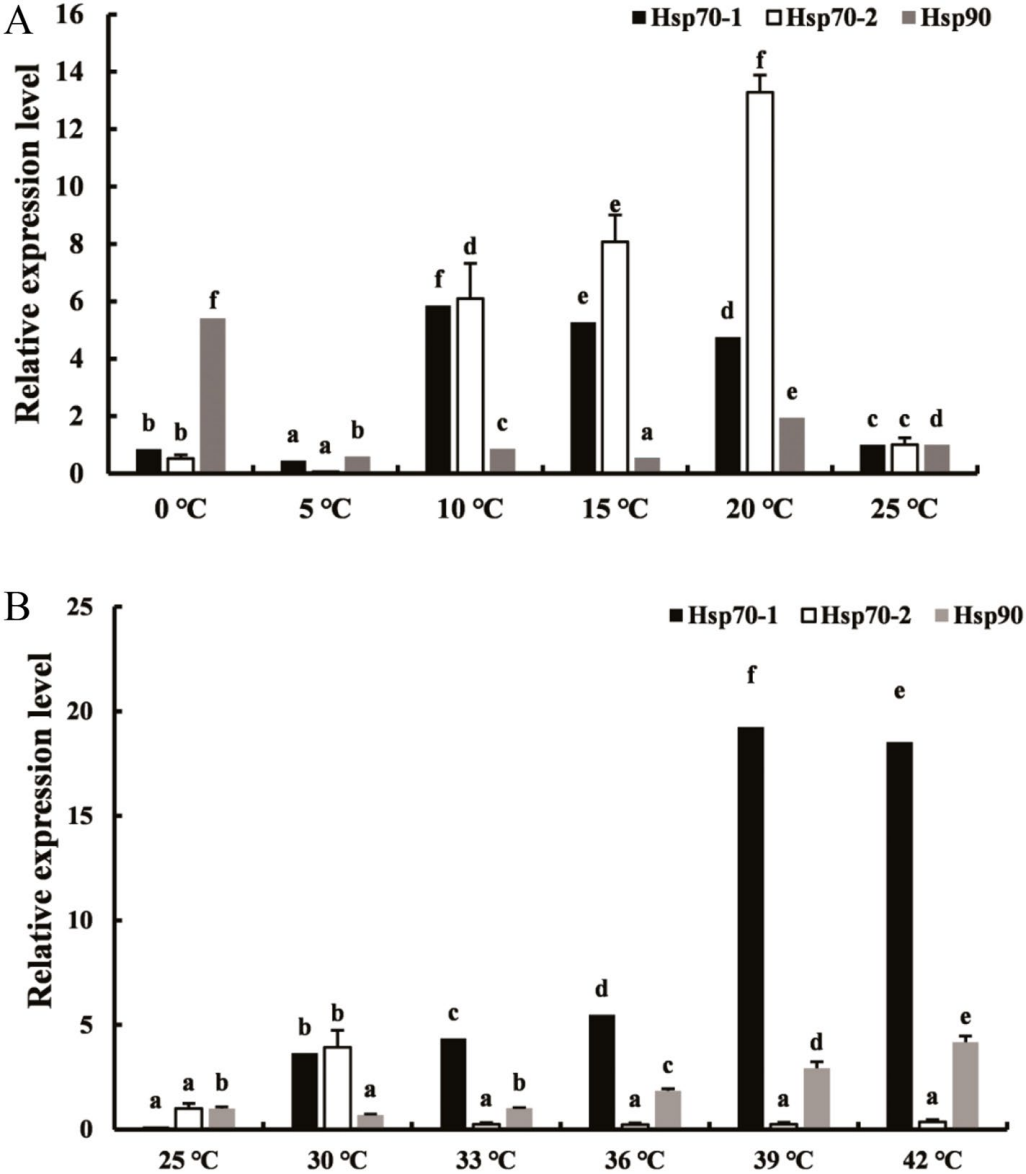
(**A**) Comparative quantitative RT-PCR analyzed of the relative expression of *TpHSP70-1*, *TpHSP70-2* and *TpHSP90* at low temperatures. Control: 25 ℃; Low heat shock temperature: 0, 5, 10, 15, 20 ℃. (**B**) Comparative quantitative RT-PCR analyzed of the relative expression of *TpHSP70-1*, *TpHSP70-2* and *TpHSP90* at high temperatures. Control: 25 ℃; High heat shock temperature: 30, 33, 36, 39, 42 ℃. Each temperature treatment was three replicates. Data were represented as the mean ± SD (n = 200). Letters above columns indicate levels of difference significance at *P* < 0.05. The same letters are not significantly different, *P* > 0.05.

The expression patterns of *TpHSP90* and *TpHSP70* mRNA were examined at low temperatures. Expression of *TpHSP70-1* gene peaked at 10℃, where it was 6.05 times higher than in the control group. The *TpHSP70-2* gene peaked at 20℃. Finally, *TpHSP90* gene peaked at 0 ℃, but no differences were observed between 5, 10, 15 and 25 ℃ (Fig. 5A). At high temperatures, expression of *TpHSP70-1* gene peaked at 39 ℃ (Fig. 5B) where expression was 19.73 times higher than in the control. The expression of *TpHSP70-2* gene peaked at 30 ℃, whereas at other temperatures expression was the same as in the control. Finally, *TpHSP90* gene expression peaked at 42 ℃ (4.17 times higher than in the control group).

## Discussion

In the present study, we cloned the three full-length cDNAs of *HSP70-1*, *HSP70-2* and *HSP90* genes and evaluated their expression in response to thermal stress, in the hope to understand how they help withstand extreme temperatures. Our results show that *TpHsp70-1* plays the most important role at low temperatures, whereas at high temperatures (heat stress), both *TpHsp70-1* and *TpHsp90* are important. Expression of HSPs helps organisms to adapt to high temperatures as shown by many studies, e.g., in *D. melanogaster*^19^, *S. exigua*^30^, *T. cinnabarinus*^24,28,31^ and *P. citri*^23,32^.

Under cold stress conditions, we found that *TpHSP70-1* and *TpHSP70-2* failed to express, consistent with *Hsp70* expression results in *T. cinnabarinus*^24,28^, *P. citri*^24^ and *D. melanogaster*^19^. This suggests other mechanisms in *T. putrescentiae* to help resist low temperatures, such as the synthesis of trehalose, polyols and other small molecules, antioxidant reactions and production of other heat shock proteins^24^.

The expression of *T. putrescentiae TpHSP70-1* and *TpHSP90* genes was up-regulated at higher temperatures and improved heat resistance. In particular, the relative mRNA expression level of *TpHSP70-1* at 39 ℃ was 19.03 times higher than the control group. The survival of *Bactrocera tryoni*’s eggs and larvae at 46 ℃ is significantly lower than the control^33^ and it has been suggested that exposure of *Ceratitis capitata* to sublethal temperatures of 42 ℃ for 1 h enhanced heat resistance^34^. The heat resistance of *Anastrepha suspensa* raised at 30 ℃ was higher than in those raised at 20 ℃^35^. Lastly, after a short period of high temperature stress, survival time was prolonged in *Cydia pomonella* exposed at lethal temperatures^36^. The synthesis of heat shock protein begins to decline after a certain threshold^32^. We found that *TpHSP70-1* expression was highest at 39 ℃ and decreased at 42 ℃, which may reflect proximity to that threshold^23,37^. Therefore, heat shock proteins can protect biological cells only to a certain extent^37^: induced high expression of heat shock proteins can improve insect heat resistance, but this expression affects the synthesis of other proteins in the insect body, which results in shortened life span and reduced fertility^27,38^. Indeed, our results show that the number of eggs laid by *T. putrescentiae* decreased significantly after exposure to temperatures above 39 ℃.

Heat shock protein genes are also involved in normal physiological activities and in fertility. These include the folding of new peptide chains to form mature proteins, the formation of gametes and cell differentiation^39^. We show that the transcription and expression level of the *HSP90* gene of *T. putrescentiae* depends on the developmental stage, being higher in the larval stage. This indicates that this gene is also involved in the regulation of growth and development. Expression of *Hsp70* fluctuated with the development period, suggesting its involvement in normal physiological activities and reproductive development.

Finally, the mRNA expression level of heat shock protein genes in the female adult was higher than in the male, consistent with *Grapholita molesta* results^27^, and hinting that the ability to cope with environmental temperature stress is higher in females.

In conclusion, new *TpHSP90*, *TpHSP70-1*and *TpHSP70-2* genes sequences were isolated from *T. putrescentiae,* and their phylogeny with other mites was inferred. Their expression levels varied with the developmental stages and the highest expression observed was in the immature mite, suggesting that *TpHSPs* genes are involved in the regulation of growth and development. These three *TpHSPs* genes are important for *T. putrescentiae* to defend against temperature and are closely related to mortality. Our study helps to understand the resistance of mites and other insects to environmental stress, and guides *T. putrescentiae* management by using different temperatures in crops.
